# Interactive Online Learning for Attending Physicians in Ultrasound-guided Central Venous Catheter Insertion

**DOI:** 10.7759/cureus.1592

**Published:** 2017-08-22

**Authors:** Sylvain Boet, Calvin Thompson, Michael Y Woo, Debra Pugh, Rakesh Patel, Pavithra Pasupathy, Asad Siddiqui, Ashlee-Ann Pigford, Viren N Naik

**Affiliations:** 1 Anesthesiology and Pain Medicine, The Ottawa Hospital, The University of Ottawa; 2 The Ottawa Hospital; 3 Ottawa Hospital Research Institute, The Ottawa Hospital; 4 Anesthesiology, The Hospital for Sick Children, Toronto, Canada; 5 Department of Anesthesia, University of Toronto; 6 Royal College of Physicians / University of Ottawa, The Ottawa Hospital / University of Ottawa

**Keywords:** physician online training, ultrasound guided central venous catheter insertion, physician education, knowledge acquisition, knowledge retention

## Abstract

Evidence has demonstrated that the use of dynamic ultrasound guidance (USG) for central venous catheter (CVC) significantly decreases attempts, failures, and complication rates. Despite national organizations recommending the use of USG and its increasing availability, USG is used inconsistently and non-uniformly. We sought to determine if an online training module for CVC insertion with ultrasound guidance will improve acquisition and long-term retention of knowledge and skills for attending physicians. Participants were tested for declarative knowledge and skills on a simulator (pre-test) for ultrasound-guided CVC insertion at baseline. They then completed an online learning module followed by an immediate post-test and a six-month retention test. There were 16 attending physicians who participated in the study. The CVC training module increased declarative knowledge acquisition and retention. No significant difference in simulated CVC performance was found over the three time points.

## Introduction

Central venous catheters (CVC) are inserted by multiple medical practitioners both electively and urgently [1]. Traditionally, CVCs have been inserted using a technique utilizing only external anatomic landmarks. The success rate is highly dependent on physician experience and knowledge of the underlying anatomy. This landmark technique has a significant complication rate that varies from 2%-19% and may be as high as 40% if cannulation failure is included [2]. Evidence has demonstrated that the use of dynamic ultrasound guidance (USG) for CVC significantly decreases attempts, failures, and complication rates by 57%-78% when compared to using the landmark technique [3]. USG-CVC is also faster to insert than traditional landmark techniques [4]. As a result, a number of medical societies (i.e., the American College of Chest Physicians, the British National Institute of Clinical Excellence, the Agency for Healthcare Research and Quality, and the College of Physicians and Surgeons of Ontario Patient Safety Review Committee) have issued guidelines in support for real-time ultrasound use with CVC insertion.

Despite national organizations recommending the use of USG and its increasing availability, USG is used inconsistently and non-uniformly. A recent survey of cardiovascular anesthesiologists in North America found that 66% of respondents never or rarely used ultrasound for CVC insertion and only 15% used it systematically [2]. Continuing professional development is effective to learn USG-CVC, but mannequin-based training may be cumbersome and challenging to organize [5]. Online USG-CVC may be appealing but does not allow the practice of skills.

Our study’s USG-CVC intervention focuses on the continuing professional development of the faculty and aims to train attending physicians using an online learning module. We aimed to measure the effect of an online CVC learning module on (i) USG-CVC declarative knowledge acquisition and retention, as measured by a written test, and (ii) CVC skill performance on a part-task trainer for CVC insertion, as measured using CVC checklist and global rating scores. Skill and knowledge measurements were made before and after the educational intervention to determine the extent of learning, as well as several months after the intervention to determine potential attrition of skills.

## Materials and methods

The study was a single blinded prospective interventional repeated measures study trial with three time measurements. This trial was approved by the Ottawa Health Science Network-Research Ethics Board (OHSN-REB) at the Ottawa Hospital Research Institute, Ottawa, Canada (#20120722-01H). Written informed consent was obtained from all study participants. Each subject acted as their own control, as each underwent repeated measurements of their USG-CVC declarative knowledge and skill performance. This manuscript adheres to the Transparent Reporting of Evaluations with Nonrandomized Designs (TREND) guideline.

All attending physicians from departments of The Ottawa Hospital who routinely perform CVC insertions were invited to participate in the study on a volunteer basis. These included physicians in Anesthesiology, Critical Care, Emergency Medicine, and Internal Medicine. Investigators were excluded from participating. A member of the investigative team contacted their respective departments; the information was then forwarded to potential participants. The interested participants scheduled a study time with the research staff.

CVC declarative knowledge (a multiple choice written test) and skills (simulated insertion) were measured at three time intervals. On the day of the online CVC course, all subjects completed a demographic questionnaire and pre-test (knowledge and skills) prior to the completion of the online course. Participants then took a 2.5-hour online course based on the CAE-ICCU USG-CVC curricula (www.caeiccu.com, CAE Healthcare, Montreal, Canada) [6]. Upon completion of the course, the participants were assessed for knowledge and skills in an immediate post-test. No feedback on any of the tests was provided. Six months after the completion of the course, the participants completed a retention test (knowledge and skills).

The online CAE-ICCU curriculum for CVC insertion using USG covers all of the relevant anatomy and imaging considerations [6]. It also includes training on the 'Assessment of Central and Peripheral Vessels' including both diagnostics and intervention. Study investigators reduced the CAE-ICCU course from 5.5 hours to 2.5 hours to make the time commitment more appropriate to study participants. To ensure that participants had access to the full curricula, they were provided with a complete license upon completion of the retention test.

We used the following performance measures:

Declarative knowledge tests: The repeated global declarative knowledge test included 20 multiple-choice questions that are clinically oriented to test the multiple dimensions of USG-CVC taken from the CAE-ICCU Learning Management System.

CVC skill performance tests: Participants demonstrated USG-CVC insertion into the right internal jugular vein on a part-task trainer for CVC insertion (Central Line Man Training Package with Articulating Head, SimuLab Corporation, Seattle WA, USA) and using a L38 linear transducer (5-10MHz) with a SonoSite M-Turbo ultrasound machine (FUJIFILM SonoSite Inc., Bothell, WA, USA). The CVC mannequin included an upper torso with an articulating head. A research assistant used a built-in hidden hand pump to simulate blood flow. Performances were videotaped without audio and framed to blind the physician's identity. Two CVC blinded experts, from another university, independently assessed each test performance using established metrics for procedural skills. A modified, previously validated CVC checklist and global rating scale (GRS), excluding the item for ‘use of assistants’, were used to assess the CVC insertion [7]. After the initial independent assessment, raters met to agree on a consensus for each scored item that had more than 1 point difference between the 2 raters. The average score was used when the difference between the raters was one or less.

We assessed the following outcomes:

Declarative knowledge was measured at three distinct intervals: pre-test prior to the online course, immediate post-course test, and retention test six months after the completion of the online training. CVC skill performance was measured on a simulated part-task trainer at three distinct intervals: pre-test, immediate post-course test, retention test six months after completion of the online training.

Considering a moderate effect size of 0.5 (Cohen's d) as per common effect of educational intervention, an α risk of 0.05 and power of 0.95, a total sample size of 16 subjects was necessary to detect acquisition of knowledge and skills (i.e., one group with two measurements - pre-test and immediate post-test) and 12 subjects were necessary to detect retention of knowledge and skills (i.e., one group with three measurements - pre-test, immediate post-test, and retention test) [8].

Knowledge test scores, checklist scores, and global rating scores were analyzed to detect differences in knowledge and skills at the three time points using a one-way repeated measure ANOVA followed by a post-hoc analysis if significant.

## Results

A total of 17 participants completed day one, which included the pre-tests, training, and immediate post-tests. One participant did not complete the retention tests six months later. Our total sample size included in the analysis was 16 physicians (knowledge and CVC skill performance at pre-test, immediate post-tests, and retention test). Demographic details can be found in Table 1.

**Table 1:** Participant demographic details

Day 1, initial tests and training	
Age (years) Mean ± SD	46.3 ±10.9
Years of practice (after residency) (years) Mean ±SD	16.0 ± 13.1
Number of CVC's conducted in past 3 years (n)	6.5
Average number of CVC's supervised in past 3 years (n)	7.9
Sex M/F	8 / 8
Specialty: Anesthesia/ Internal medicine/ Other	12/2/2
Previous formal CVC training experience (n) / Didactic (n) / Workshop (n)	10/9/1
Previous CVC simulation training experience (Y/N)	0/16
Previous online CVC training experience (Y/N)	2/14
Previous simulation training experience (Y/N)	5/9
Day 2, 6-month retention tests	
Average number of days to retention (days) Mean ± SD	197.1 ± 25.6
Average number of CVC's conducted since the last session (n)	1.1
Maximum number of CVC's conducted since the last session (n)	5
Minimum number of CVC's conducted since the last session (n)	0
Participated in training since last session (n) / Didactic (n) / Other (n)	2/2/0
CVC simulation training experience since the last session (n)	0
Online CVC training experience since the last session (n)	0
Simulation training experience since the last session (n)	0

Declarative knowledge:

One-way repeated measures ANOVA for knowledge test data suggests that there is a significant difference (F(30,2)=42.0, P<0.005) between pre-test, immediate post-test, and retention test (Table 2). Post-hoc analysis (Bonferroni) suggests that participants improved significantly from pre-test to immediate post-test (p<0.005) following the CVC training module. Participants also increased from pre-test to retention test (p=0.035) but decreased from immediate post-test to retention test (p<0.005) (Table 2).

CVC skill performance:

Using the modified CVC checklist (10 points), there was no significant difference between the test phases (F(30,2)=0.77, P=0.47) (Table 2). Using the GRS for procedural skills (30 points), there was no difference between the test phases (F(30,2)=0.55, P=0.58) (Table 2).

 

**Table 2:** Outcome measures over three time points (pre-test, immediate post-test, and retention test)

	Declarative knowledge	CVC skill performance	
	Declarative Knowledge Test Data (% correct) Mean +/- SD	Modified CVC C hecklist (/10 points) Mean +/- SD	Global Rating Scale for Procedural Skills (/30 points) Mean +/- SD
Pre-Test	59.7 ± 14.1	8.8 ± 0.8	20.5 ± 6.1
Immediate Post-Test	90.0 ± 8.2	9.0 ± 0.7	22.4 ± 4.8
Retention Test (6 months)	70.6 ± 12.2	8.6 ± 1.3	21.1 ± 5.6
Statistical test performed and significance	One-way repeated measures ANOVA between pre-test, immediate post-test and retention test: p<0.005 Post-hoc analysis (Bonferroni): from pre-test to immediate post-test: p<0.005 from pre-test to retention test: p=0.035 from immediate post-test to retention test: p<0.005)	One-way repeated measures ANOVA between pre-test, immediate post-test and retention test: p=0.47	One-way repeated measures ANOVA between pre-test, immediate post-test and retention test: p =0.58

## Discussion

This study suggests that attending physicians have the capacity to learn and retain declarative knowledge from an online CVC training module but without any significant changes on simulation-based testing for CVC insertion at multiple time points. A ceiling effect may partially explain the lack of effect on the checklist score (pre-test score of 88%). However, regarding the GRS score, a ceiling effect is unlikely with a pre-test score of 68%. Although no definitive threshold of competency exists for the CVC checklist and GRS scores, the post-tests skill performance indicates room for improvement even after online training with 75% and 70% on the immediate post-test and retention GRS scores, respectively. Studies recruiting residents found comparable post-tests performance around 80% [7]. Studies recruiting attending physicians have demonstrated the effectiveness of training on CVC skills but baseline performance was often extremely poor [5,9]. However, all these studies included some level of deliberate practice, i.e., simulation practice and feedback, that was absent from our online course.

## Conclusions

Overall, our findings support the use of online CVC training to improve the CVC declarative knowledge of physicians. Deliberate practice may be necessary to complement online training to successfully reach a virtually perfect level in CVC skills for attending physicians.
